# Unveiling the interfacial electrochemiluminescence behavior of lead halide perovskite nanocrystals†

**DOI:** 10.1039/c9na00456d

**Published:** 2019-09-03

**Authors:** Linghang Qiu, Longhui Lin, Yipeng Huang, Zhiwei Lai, Feiming Li, Shuya Wang, Fangyuan Lin, Jianfeng Li, Yiru Wang, Xi Chen

**Affiliations:** Department of Chemistry, The MOE Key Laboratory of Spectrochemical Analysis & Instrumentation, College of Chemistry and Chemical Engineering, Xiamen University Xiamen 361005 China xichen@xmu.edu.cn; State Key Laboratory of Marine Environmental Science, Xiamen University Xiamen 361005 China; Shenzhen Research Institute of Xiamen University Xiamen 361005 China

## Abstract

In this study, a three-phase heterostructure interface including glassy carbon (conducting medium), CsPbBr_3_ perovskite nanocrystals (PNCs, emitter) and acetonitrile (electrolyte) is constructed for fully investigating the interfacial electrochemiluminescence (ECL) behavior of CsPbBr_3_ PNCs. We find that these interfaces serve as bridges for efficient electron–hole transfer during the ECL process. As a proof of concept, the increase of the heterostructure interface area will accordingly enhance the ECL intensity of CsPbBr_3_ PNCs. About seven-fold enhancement of the ECL intensity could be achieved when the interface area has triple-fold increase, which provides a new perspective to construct more efficient ECL systems *via* interface engineering.

## Abstract 2

Herein, a three-phase heterostructure interface including glassy carbon, CsPbBr_3_ perovskite nanocrystals and acetonitrile is constructed for fully investigating the interfacial electrochemiluminescence (ECL) behavior of CsPbBr_3_ PNCs.

## Graphical abstract

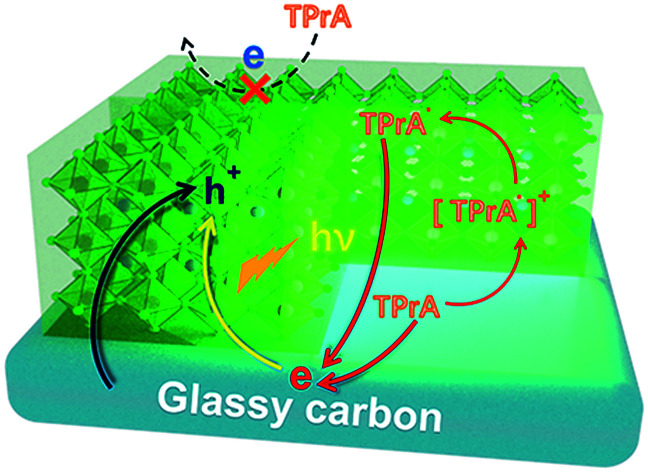

## Introduction

Lead halide perovskite nanocrystals (LHPNCs) have attracted tremendous attention due to their remarkable optical properties including high brightness, tunable emission wavelength, narrow band emission, and high defect tolerance, which are promising emitters in the field of electrochemiluminescence (ECL).^1–5^ Since the ECL phenomena of CsPbBr_3_ PNCs in dichloromethane were discovered in 2016,^6^ relevant studies have focused on the ECL behavior of perovskites. Typically, the influence of the sequence of electron and/or hole injection processes on the ECL of CH_3_NH_3_PbBr_3_ NCs was investigated.^7^ In addition, the lead-free perovskite, Cs_3_Bi_2_Br_9_ QDs was introduced to investigate the ECL activities.^8^ Furthermore, hydrogen peroxide (H_2_O_2_) was introduced as a model of reactive oxygen species (ROS) to investigate the ECL of CsPbBr_3_ PNCs.^9^ In order to achieve better ECL performance, Xue *et al.* proposed a scraping coating method to acquire high-quality CsPbBr_3_ PNC film and introduced anhydrous ethyl acetate as an anti-solvent and co-reactive agent.^10^ Recently, Li *et al.* simultaneously encapsulated CsPbBr_3_ QDs and a coreactant into an *in situ* generated SiO_2_ matrix to achieve an efficient and stable ECL.^11^ These pioneering studies demonstrate that LHPNCs could serve as a promising candidate for ECL, especially for the organic phase ECL. Unfortunately, deep insights into the corresponding ECL process of LHPNCs are still unknown.

As is well-known, the whole ECL process should satisfy three indispensable conditions:^12–15^ (1) the injection of the electron (or hole) to the emitter and the coreactant; (2) the generation of oxidative species and reductive species; (3) the combination of intermediates to produce the excited state, along with the generation of the ECL signal. In addition, the inherent optical properties of the emitter are the basic insurance of ECL response. In order to reduce the surface energy of the PNCs, ligands are generally introduced to passivate PNCs during their synthesis.^16–21^ However, the existence of flooded long-chain ligands on the PNC surface results in limited conductivity, which greatly hinders the occurrence of the first condition mentioned above. In addition, our earlier study on the CsPbBr_3_ PNC ECL also shows that the type and content of the surface ligands of CsPbBr_3_ PNCs do have a significant impact on the ECL intensity.^13^ Furthermore, the increase of the film layer thickness also reduces the interface conductivity, which greatly affects the ECL response. Correspondingly, in order to improve the conductivity of the film layer, previous reports proposed that efficient charge transport can be achieved *via* crystallized 2D perovskites in a vertically orientated way.^22,23^ Li *et al.* added carbon nanotubes into the perovskite film to reduce the interface resistance and promote the charge conduction.^24^ Besides the field of ECL, similar interfacial problems also exist in the construction of photoelectric devices such as perovskite light-emitting diodes (peLEDs).^25–31^ The same obstacle of conductivity limitation prevails in both fields, which may lead to some common rethinking of the PNC film fabrication. Apparently, all these studies reveal that the ECL characteristics of CsPbBr_3_ PNCs also depend heavily on interfacial structures. However, limited research has been conducted to explore the ECL characteristics at the interfaces. Hence, unveiling the interfacial ECL characteristics of CsPbBr_3_ PNCs is quite important for a better understanding of the ECL process and therefore for constructing more efficient ECL systems.

**Fig. 1:**
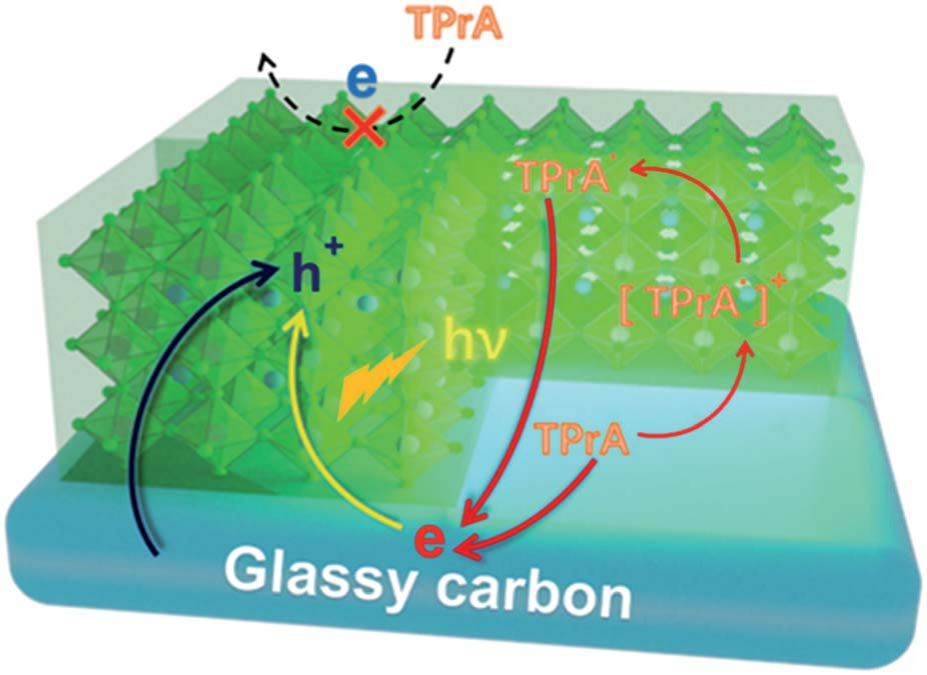
Schematic of ECL reactions on the three-phase interface consisting of glassy carbon, CsPbBr_3_ PNCs and acetonitrile (containing 10 mM TPrA).

Herein, in view of the importance of the study of interfacial ECL characteristics, a three-phase interface of electrode–emitter–electrolyte has been established by a partially uncovered CsPbBr_3_ PNC film on a glassy carbon electrode (GCE) surface (Fig. 1). The constructed three-phase interfaces enable the interfacial electrons (or holes) to be effectively injected into the perovskite layer. As illustrated in Fig. 1, the strong reducing intermediate generated from the coreactant, tripropylamine (TPrA), reacts with the strong oxidant of 
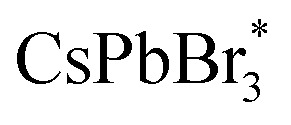
 PNCs, to produce the excited state of CsPbBr_3_ PNCs and then generate the ECL at the constructed interfaces. Accordingly, the ECL response will be enhanced with the increase of the three-phase interfaces. Moreover, enhanced ECL signal could also be observed on a thicker perovskite film due to the larger area of the exposed three-phase interface since the thickness of the film at the micron level increases with increasing grain boundary, which gives new insights in interface engineering for perovskite films.

## Experimental

### Methods

#### Chemicals and reagents

Cesium carbonate (Cs_2_CO_3_) and alpha,alpha′-dibromo-*p*-xylene (Dbpx) were purchased from Energy Chemical Reagent Co., Ltd (Shanghai, China). Oleylamine (OAm), lead stearate (PbSt_2_) and tripropylamine (TPrA) were obtained from Aladdin (Shanghai, China). 1-Octadecene (ODE) was purchased from Alfa Aesar (China). Acetonitrile was purchased from Shanghai Chemical Reagent Co., Ltd. All reagents were of analytical grade expect OAm and ODE, which were redistilled.

#### Apparatus and characterization

Ultraviolet absorption spectra were recorded using a Shimadzu UV-2550 and an F-7100 spectrophotometer (Hitachi, Japan) was employed to collect fluorescence emission spectra. The morphologies of samples were observed by scanning electron microscopy (SEM, Hitachi S4800, Japan) and transmission electron microscopy (TEM) with a JEM-1400 microscopy system (JEOL, Japan) at an acceleration voltage of 120 kV. AFM images were acquired using a Cypher S atomic force microscope (AFM, Oxford instruments, Britain) in contact mode. X-ray diffraction (XRD) spectra were recorded using a Rigaku Ultima IV instrument (Kuraray, Japan) with a Cu target (*λ* = 1.54051 Å) operated at 40 kV and 15 mA. The ECL response was recorded with a model MPI-A ECL analyzer from Xi'an Remax Electronic Science & Technology Co. Ltd (Xi'an, China) combined with a CHI 660C electrochemistry workstation from Shanghai CH Instruments (Shanghai, China) to perform electrochemical measurements simultaneously. The photomultiplier tube (PMT) was biased at 600 V. A conventional three-electrode system was introduced in the process, which comprised a modified GCE (*Φ* = 3 mm) as the working electrode, Ag/AgCl (saturated KCl solution) as the reference electrode, and a platinum wire as the counter electrode.

#### Preparation of CsPbBr_3_ PNCs

CsPbBr_3_ PNCs were prepared by a simple one-step heating method after optimization according to a previous report.^32^ Specifically, ODE (5 mL), OAm (0.75 mL), PbSt_2_ (0.05 mmol), Dbpx (0.2 mmol) and Cs_2_CO_3_ (0.05 mmol) were sequentially added into a 25 mL two-neck flask. The mixture was heated from room temperature to 150 °C under intense agitate and then transferred to an ice bath for immediately cooled down. The product was centrifuged at 10 000 rpm for 10 min to discard the supernatant, and then the precipitates were washed with hexane two times by centrifugation. Finally, the precipitates were dried in a vacuum drying oven for subsequent use.

#### Preparation of CsPbBr_3_ PNCs|GCE

A GCE was polished with 0.3 μm alumina slurry, ultrasonically washed using ultrapure water and then dried in a nitrogen atmosphere. 5 μL of 0.1 g mL^−1^ colloidal CsPbBr_3_ PNC slurry dispersed in hexane was dropped on the GCE surface to construct a CsPbBr_3_ PNC film for further ECL measurement.

## Results and discussion

**Fig. 2:**
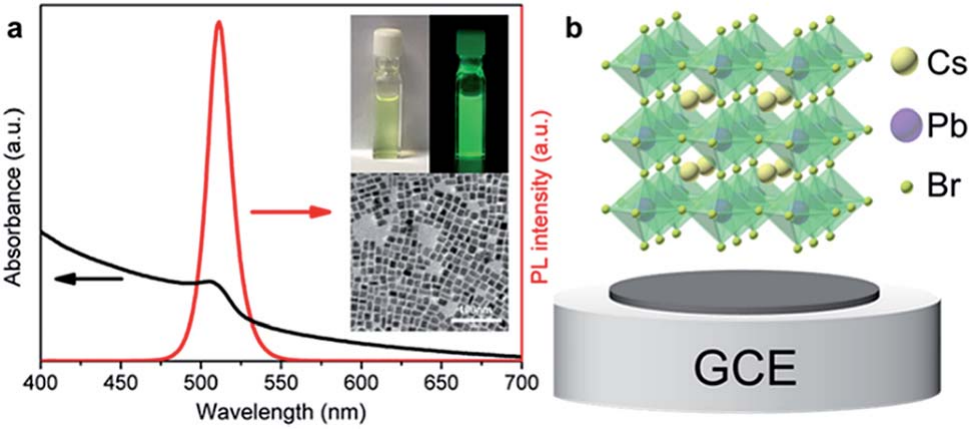
(a) PL (red line) and UV-vis absorption (black line) spectrum of CsPbBr_3_ PNCs dispersed in hexane. Inset: the upper one shows CsPbBr_3_ PNCs under visible light (lamp off) and UV light (365 nm, lamp on); the bottom one shows the TEM image of CsPbBr_3_ PNCs. (b) Schematic diagram of CsPbBr_3_ PNCs|GCE.

The obtained CsPbBr_3_ PNCs display a distinct excitonic absorption peak around 504 nm and a sharp photoluminescence (PL) peak at 511 nm with a full width at half-maximum (FWHM) of 18 nm, demonstrating a bright green emission under 365 nm UV light (Fig. 2a). In addition, the CsPbBr_3_ PNCs present good monodispersity with an average size of 13 nm and could be well dispersed in hexane, which is beneficial for the CsPbBr_3_ PNC film fabrication. In this study, a CsPbBr_3_ PNC film was fabricated by drop-coating 5.0 μL of colloidal CsPbBr_3_ PNC slurry (0.1 g mL^−1^) on a GCE surface. The film was then left to dry naturally (Fig. 2b) for the subsequent ECL investigation. CsPbBr_3_ PNCs were dropped on the electrode surface with a high concentration compared with those reported in the literature.^6,7,9^ In addition, the relatively slow process of solvent evaporation with the appropriate amount of ligands on CsPbBr_3_ PNCs makes it relatively easier to fabricate a compact film. The scanning electron microscopy (SEM) image shown in Fig. S1a† illustrates that the CsPbBr_3_ PNC film on the GCE is quite compact with a micron level thickness. The surface roughness of the CsPbBr_3_ PNC film was further characterized using atomic force microscopy (AFM). As shown in Fig. S2,† the AFM image clearly reveals that the surface roughness is around 2 nm. These results confirm the compact and pin-hole free surface of the CsPbBr_3_ PNC film (Fig. S2a†), which is quite important for the three-phase interface construction.

**Fig. 3:**
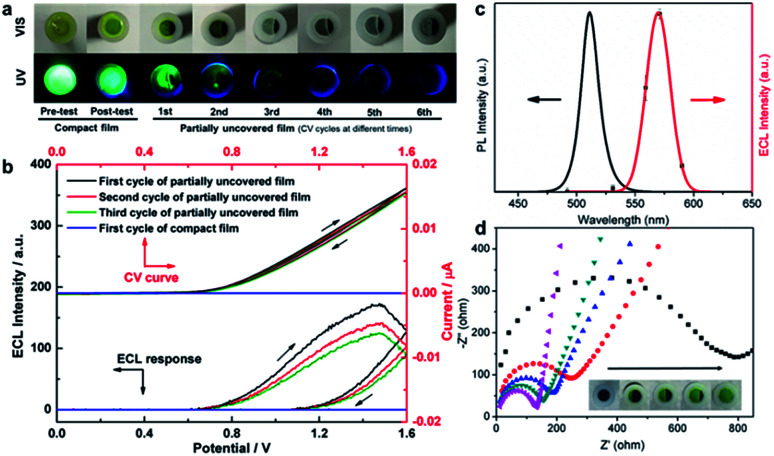
ECL response of CsPbBr_3_ PNCs|GCE in acetonitrile containing 10 mM TPrA under continuous cycling scans from 0 V to 1.6 V. The scan rate was set at 0.05 V s^−1^. (a) Pattern changes of CsPbBr_3_ PNCs|GCE before and after several CV cycles. Top row images were taken under visible light, and bottom row images were taken under 365 nm UV light. (b) The ECL emission and cyclic voltammograms from the compact film and partially uncovered film under cycle scans for one to three cycles, respectively. (c) PL (black line, left) and ECL (red line, right) spectra of CsPbBr_3_ PNCs|GCE. (d) EIS curve of CsPbBr_3_ PNCs|GCE with different areas of exposed glassy carbon.

In this study, ECL tests were performed in acetonitrile solution containing 10 mM TPrA. In the ECL process, acetonitrile was introduced as the electrolyte on account of its favorable conductivity and the low solubility of CsPbBr_3_ PNCs. Owing to the presence of the insulating oleylamine (OAm) ligands on the surface of CsPbBr_3_ PNCs, electrons are severely blocked in the electrolyte, which impedes electron injection into the CsPbBr_3_ layer and the GCE is covered with CsPbBr_3_ PNCs in a compact manner. This point could be confirmed well from the undetectable current and ECL signal using the GCE with a compactly covered film of CsPbBr_3_ layer (Fig. 3b). A control experiment was conducted in the absence of TPrA (Fig. 3b). There is also no ECL response with the compact film. Surprisingly, the electrolytic current and ECL signal obviously increased when the CsPbBr_3_ film was partially uncovered by gently scraping the compact film to remove the partial-film (Fig. 3b). In addition, compared with the compactly covered film, the electrochemical impedance spectrum (EIS) reveals obvious impedance decrease when a small amount of the CsPbBr_3_ film was removed to expose the GCE surface (Fig. 3d). This result indicates that the formation of the interface among CsPbBr_3_ PNCs, glassy carbon and acetonitrile (containing TPrA) opens up the electron or hole transmission channel, and thus triggers the whole reaction. After the ECL measurement, the CsPbBr_3_ film remains compact (Fig. S1b†), indicating that the electrolyte has failed to infiltrate into the film and the ECL signal merely generates on the interfaces.

As a classical coreactant, the addition of TPrA significantly enhances the anodic ECL signal. In a specific reaction process, with a continuous positive scan from 0 V to 1.6 V in the presence of TPrA, CsPbBr_3_ is transformed to a strong oxidizing intermediate [CsPbBr_3_]^+^˙ and TPrA is transformed to a strong reducing intermediate, TPrA˙, respectively. TPrA˙ is the electron donor at the three-phase interface, and the partially uncovered glassy carbon interface acts as an electron transmission bridge, which combines with [CsPbBr_3_]^+^˙ to generate an excited state 
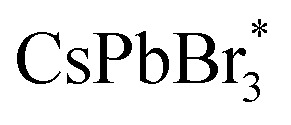
 and finally produce the ECL. Without the three-phase interface, electron transmission is blocked, which inhibits ECL generation.CsPbBr_3_ − e^−^ → [CsPbBr_3_]^+^˙TPrA − e^−^ → TPrA˙ + H^+^
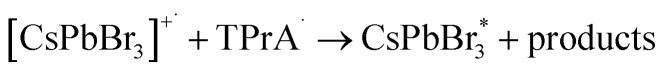

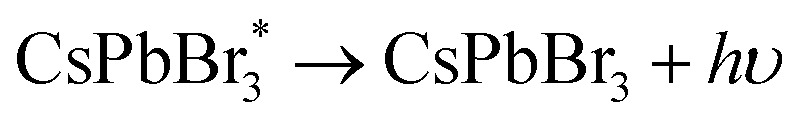


The ECL emission peak was found to be 569 nm with a FWHM of 26 nm, showing a red shift of 58 nm compared with the PL emission of CsPbBr_3_ PNCs (Fig. 3c). Such a red shift illustrates that there is a difference between the ECL and PL processes. In general, ECL performance is closely related to the surface state which may lead to a red shift.^33^ On one hand, the impact of ligands cannot be ignored as they reflect the surface properties of CsPbBr_3_ PNCs and influence the electron transfer process. On the other hand, the secondary growth of CsPbBr_3_ PNCs on the surface leads to an increase in the size of CsPbBr_3_ PNCs. It changes the surface state of the CsPbBr_3_ PNCs and causes the ECL red shift. Since the ECL experiments were carried out in acetonitrile and the potential was repeatedly applied, as shown in Fig. 3a, the color of the CsPbBr_3_ PNC film gradually changed from green-yellow to white with increase in the cycle number in the cyclic voltammetry (CV) measurements. The corresponding ECL responses also show an attenuated tendency (Fig. 3b). Following the verification of the CsPbBr_3_ film, the frequent re-construction of the surface may cause response attenuation, relative to the dissolution and recrystallization processes because of the low solubility of CsPbBr_3_ in acetonitrile.

**Fig. 4:**
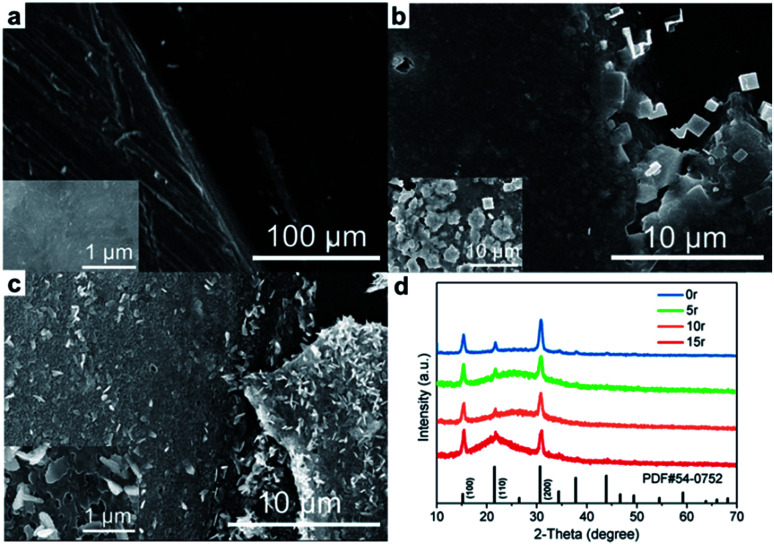
SEM image of CsPbBr_3_ PNCs|GCE with half area exposure of the electrode surface under cyclic voltammetry scanning steps from 0 to 1.6 V for (a) 0; (b) 5; (c) 10 cycles. Insets show surface conditions around the boundary between the exposed glassy carbon interface and CsPbBr_3_ PNC film. (d) XRD patterns of CsPbBr_3_ PNCs subjected to CV cycle steps from 0 to 1.6 V for 0, 5, 10, 15 times, respectively. All scan rates are set at 0.05 V s^−1^.

In order to verify the presumption that CsPbBr_3_ PNCs undergo reconstruction on the electrode surface, SEM images of the CsPbBr_3_ film before and after ECL measurements were observed. As shown in Fig. 4a–c, a smooth surface could be found for the pristine film, while the bulk crystals at the micron level appeared at the boundary after 5 continuous cycles of the potential scan from 0 V to 1.6 V. The X-ray diffraction (XRD) patterns of the CsPbBr_3_ films after CV scans show no new diffraction peaks compared with that of the pristine CsPbBr_3_ film (Fig. 4d). The typical diffraction peaks at 15.3°, 21.7° and 30.7° correspond to the (100), (110) and (200) crystal facets of the cubic CsPbBr_3_ nanocrystals (PDF#54-752) in these samples, which indicates that the CV process is not related to the phase transformation of CsPbBr_3_. Therefore, the reconstruction of CsPbBr_3_ on the surface is only correlated with the change in crystal size. To further prove this observation, the bromine content in the electrolyte after the CV scan was determined using the reported halide exchange strategy.^4^ In the determination, after the CV scan (5 cycles) the electrolyte was added to the CsPbI_3_ PNC dispersion (Fig. S4†). Eventually, the PL peak exhibited a significant blue shift, indicating that Br^−^ was indeed partially dissolved in the electrolyte during the ECL reaction. For comparison, the electrode covered by the CsPbBr_3_ film was soaked in acetonitrile for 5 min without galvanization (the time period is equivalent to the time for CV scan). When pure acetonitrile was directly added to the dispersion solution, a small red shift of the PL peak of CsPbI_3_ PNCs could be observed since acetonitrile acts as an anti-solvent to agglomerate CsPbI_3_ PNCs in toluene. In addition, results from the Energy Dispersive Spectrum (EDS) experiment also confirm the loss of bromine (Fig. S5†). With the increase of cycle scan number, the percentage of Br atoms at the three-phase interface decreases continuously. The absence of Br^−^ verifies the regrowth of the layer in the reaction. In addition, the small amount of CsPbI_3_ PNCs on the electrode surface compared with a relatively large amount of electrolyte also leads to partial ligand dissolution.^34^ Therefore, obvious aggregation results in secondary growth and micron scale crystals. With continuous CV scanning, the CsPbBr_3_ PNC film undergoes fragmentation and pore formation gradually. After 10 cycles, plenty of lamellae appearing on the film surface indicate the collapse of the perovskite structure and the continuous decrease of the corresponding ECL response. From the observation of the film structure during the ECL process, it is found that CsPbBr_3_ PNC films are always subject to the trend of constant remodeling and disintegration. Therefore, it is necessary to adopt bulk perovskites or a thicker film to achieve relatively enduring and stable ECL signals due to the stability of the perovskite in the electrolyte and the resistance to the potential erosion.

**Fig. 5:**
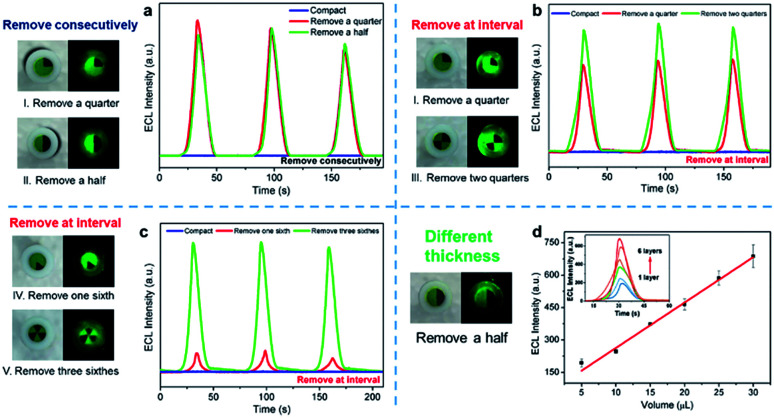
Three-phase interface construction and corresponding ECL response. The interfaces were constructed with two film removal models: (a) consecutive removal; (b and c) removal at intervals. (d) Different thicknesses by multi-layer drop-coating from 1 layer to 6 layers with removing a half of the film. All experiments under continuous cycling scans from 0 to 1.6 V in acetonitrile, the scan rate was 0.05 V s^−1^.

In order to investigate the effect of the three-phase interface exposure area on the ECL response, a three-phase interface was fabricated semi-quantitatively by properly uncovering the perovskite film from the same electrode surface (Fig. 5). Different removing patterns for the three-phase interface construction are described in Fig. S6.† The corresponding electrochemical active area (*A*) could be estimated following the previous report (Table S1†).^35^ In the process of continuous removal, we found that the ECL response on removing a quarter of the CsPbBr_3_ PNC film (pattern I) is close to that of half removal (pattern II, Fig. 5a). However, EIS curves, as shown in Fig. S7,† reveal that the impedance decreased obviously with the exposure area increase of the GCE. The electrode conductivity increase means that there are more electrons taking part in the ECL reaction. In fact, whether it is the quarter-removed or the half-removed film (pattern I/pattern II), the effective exposure area of the three-phase interface is almost the same due to the same length of the two radii and the thickness of the layer (5 μL CsPbBr_3_ PNCs coated). Considering the ECL response in the continuous removal mode (Fig. 5a), it is manifested that the number of electrons involved in the reaction on the three-phase interface is limited. The extra electrons do not participate in the ECL reaction, but cause increase in the conductivity. On the other hand, in the mode of removing at intervals (Fig. 5b and c), ECL response is enhanced for the two quarters-removed film (pattern III) compared with the quarter-removed film (pattern I) as shown in Fig. 5b. Moreover, the exposed area of the two quarters-removed film on the GCE surface (pattern III) is the same as the half-removed film (pattern II) while the effective area of the three-phase interface is different. That is, the former interface involves a length of about four radii and a thickness of one layer. It is thus clear that the ECL response of the perovskite film has no significant relationship with the exposed area of the GCE surface, but is closely related to the effective three-phase interface. Similarly, as shown in Fig. 5c, comparing the three-sixth-removed film (pattern V) by interval removal with the one sixth-removed film (pattern IV) the strength increases by nearly sevenfold, which further reveals the importance of the three-phase interface exposure. In addition, as shown in Fig. S8,† the ECL intensity is in nearly direct proportion to the number of three-phase interfaces which reiterates the interface model for the ECL of CsPbBr_3_ PNCs.

In addition, the ECL responses show a linear increase trend as the boundary thickness of the CsPbBr_3_ PNC film prepared by multi-layer drop-coating along with the half-removed film as shown in Fig. 5d. The sectional view of the electrode with different layers is recorded in the SEM images (Fig. S9†). Combined with the interfacial behavior of the ECL reaction as aforementioned, we speculate that the glassy carbon (electrode) injects holes into the CsPbBr_3_ PNC film and produces sufficient TPrA˙ radicals (coreactant in the electrolyte), thereby the thicker CsPbBr_3_ PNC film (emitters) produces positive radicals [CsPbBr_3_]^+^˙ to accept more electrons from TPrA˙ radicals on the three-phase interface, which ultimately increases the ECL response.

## Conclusions

Previous studies on the ECL of perovskites have always focused on electrochemical processes rather than the electrode interfaces. In this study, we revealed the interfacial ECL characteristics of perovskite nanocrystals in acetonitrile, and properly established the relationship between the construction of a three-phase interface and ECL response. We verified that with the increase of three-phase interfaces, that is, the more interfaces meeting with the essential conditions of ECL, stronger ECL signals could be obtained. This work provides a new idea to explore the ECL characteristics of perovskite, presenting a unique angle for interface engineering construction of perovskite film.

## Conflicts of interest

There are no conflicts to declare.

## Supplementary Material

NA-001-C9NA00456D-s001
