# Supplementary material for: Unveiling the interfacial electrochemiluminescence behavior of lead halide perovskite nanocrystals
Source: Nanoscale Adv. 2019 Sep 3;1(10):3957–62. doi: 10.1039/c9na00456d (PMC9417726; doi:10.1039/c9na00456d)
Supplement: NA-001-C9NA00456D-s001 [file NA-001-C9NA00456D-s001.pdf]

Electronic Supplementary Material (ESI) for Nanoscale Advances.
This journal is © The Royal Society of Chemistry 2019

Electronic Supplementary Information

Unveiling Interfacial Electrochemiluminescence Behavior of Lead Halide Perovskite Nanocrystals

Linghang Qiu,‡[a] Longhui Lin,‡[a] Yipeng huang,[a] Zhiwei Lai,[a] Feiming Li,[a] Shuya Wang,[a] Fangyuan Lin,[a] Jianfeng Li,[a] Yiru Wang,[a] Xi Chen[a,b,c]*

[a]Department of Chemistry and the MOE Key Laboratory of Spectrochemical Analysis & Instrumentation, College of Chemistry and Chemical Engineering, Xiamen University, Xiamen 361005, China

[b]State Key Laboratory of Marine Environmental Science, Xiamen University, Xiamen 361005, China

[c]Shenzhen Research Institute of Xiamen University, Xiamen 361005, China

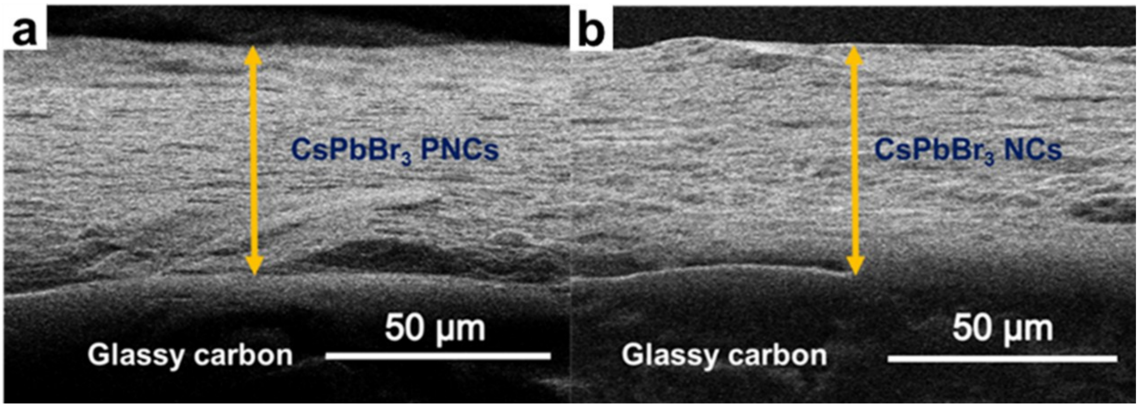

Fig. S1 SEM image of properly coated $CsPbBr_3$ PNCs film on glassy carbon substrate. (a) Fresh film without CV cycle; (b) under cyclic voltammetry scanning steps from 0 to 1.6 V for 5 times.

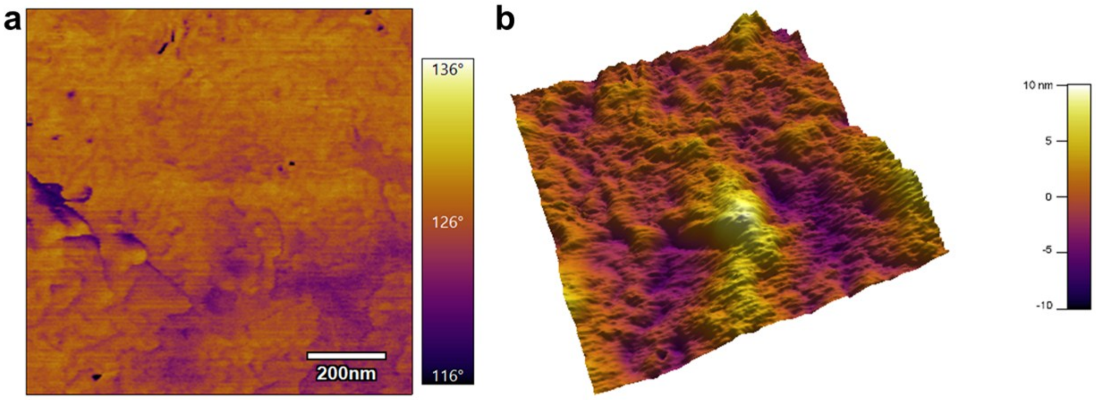

Fig. S2 AFM image of properly coated $CsPbBr_3$ PNCs film on glassy carbon substrate. (a) the phase retrace image; (b) the 3D AFM image.

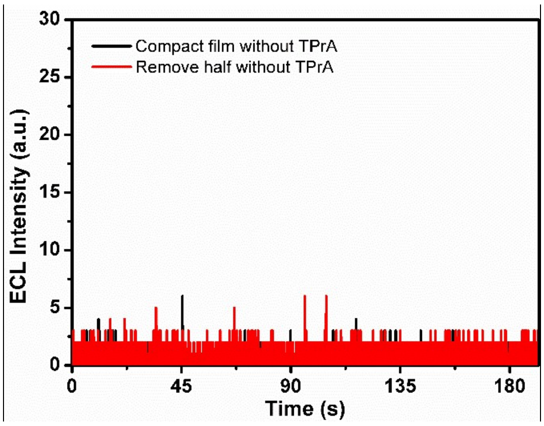

Fig. S3 The ECL response of $CsPbBr_3$ PNCs without the presence of TPrA.

Only annihilation ECL may occur on the electrode surface without the presence of TPrA, but the signal is too weak to detect as Fig. S3 shows (red line). The annihilation ECL just relates with the electron-hole transfer between the electrode and the emitter. The three-phase interface does not provide new transfer way between electrode and the emitter, because the interface aims to provide the platform for the electrolyte, the grassy carbon and the emitter to transfer electron and/or hole. Therefore, it benefits for the process in which coreactant is involved.

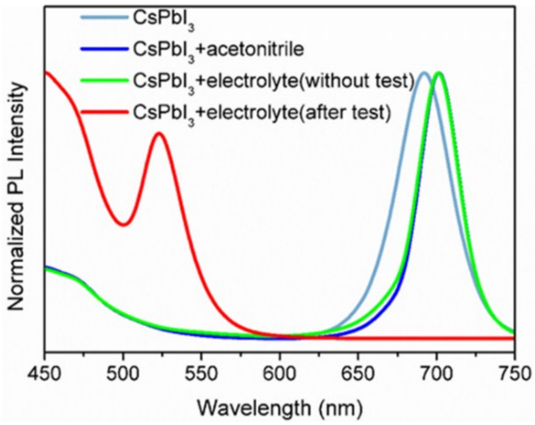

Fig. S4 PL spectra for halogen exchange strategy to determine bromine content. $CsPbI_3$ PNCs was disperse in toluene, the electrolyte refer to acetonitrile contained 10mM TPrA, and the test was under cyclic voltammetry scanning steps from 0 to 1.6

V for 5 times.

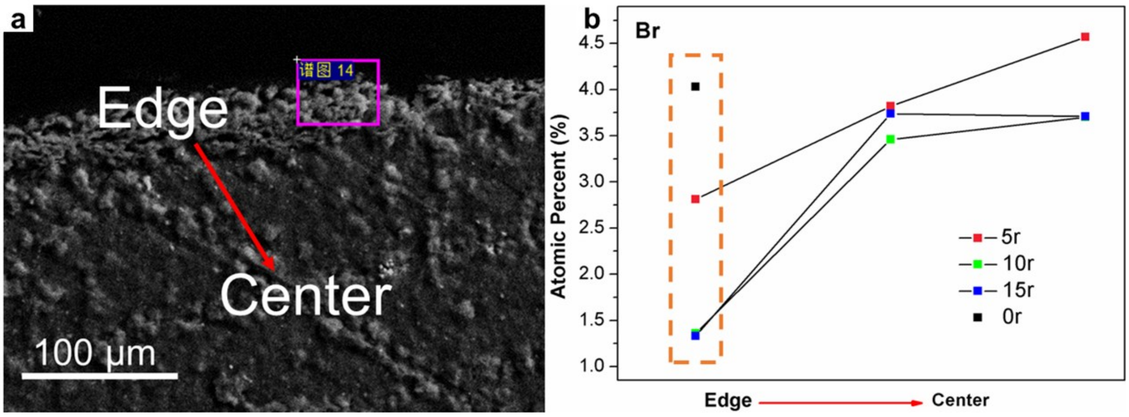

Fig. S5 (a) EDS area of the $CsPbBr_3$ PNCs|GCE; (b) corresponding distribution of Br element from the edge of $CsPbBr_3$ PNCs film to the center of the film.

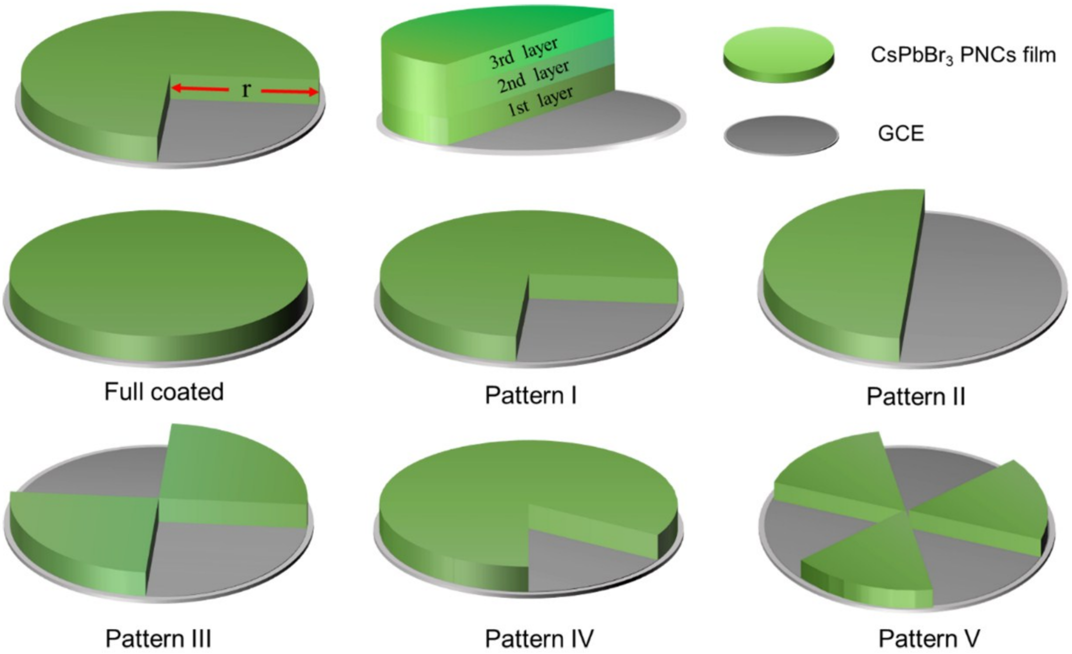

Fig. S6 Schematic of different removing patterns for three-phase interface construction.

Remove pattern	Remove area	A for different Remove mode (cm^2)
I	A quarter	0.012
II	A half	0.023
III	Two quarters	0.022
IV	One sixth	0.007
V	Three sixthes	0.023

Table S1. The electrochemical active area (A) for different remove mode corresponding to Fig. 4.

A of partial coated electrode was determined in 5 mM Fe^{2+}/Fe^{3+} in 0.5 M H_2SO_4 at different v as the Randles-Seveik equation[1].

$$i_p = 2.69 \times 10^5 n^{3/2} A D^{1/2} v^{1/2} c$$

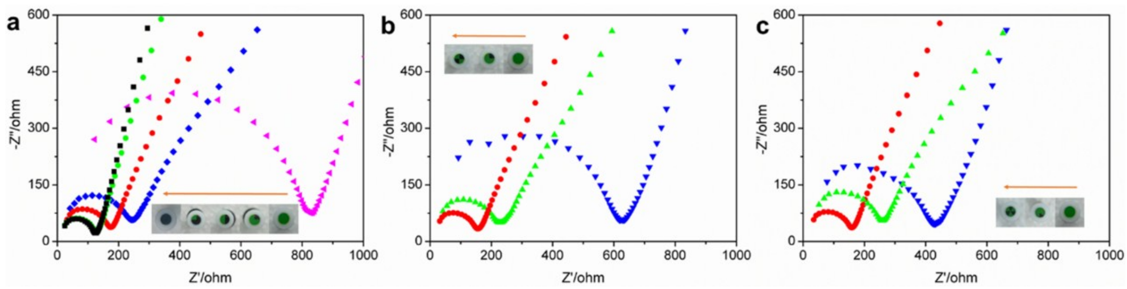

Fig. S7 EIS curve of different remove mode. (a) Remove consecutively from completely covered to entirely removed (remove a quarter per time), blue dot: pattern I; red dot: pattern II. (b) Remove at interval, green dot: pattern I; red dot: pattern III. (c) Remove at interval, green dot: pattern IV; red dot: pattern V.

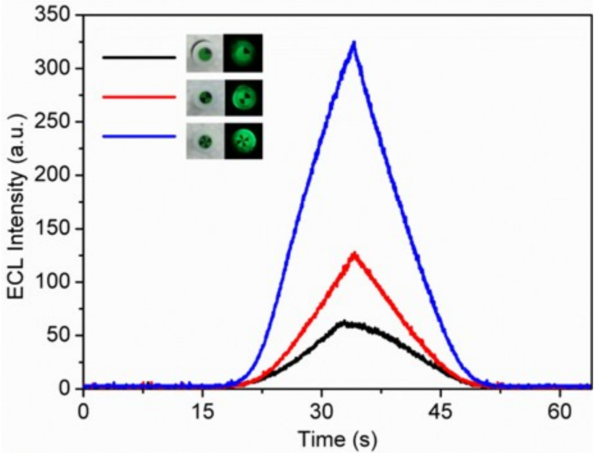

Fig. S8 ECL response of different coating patterns.

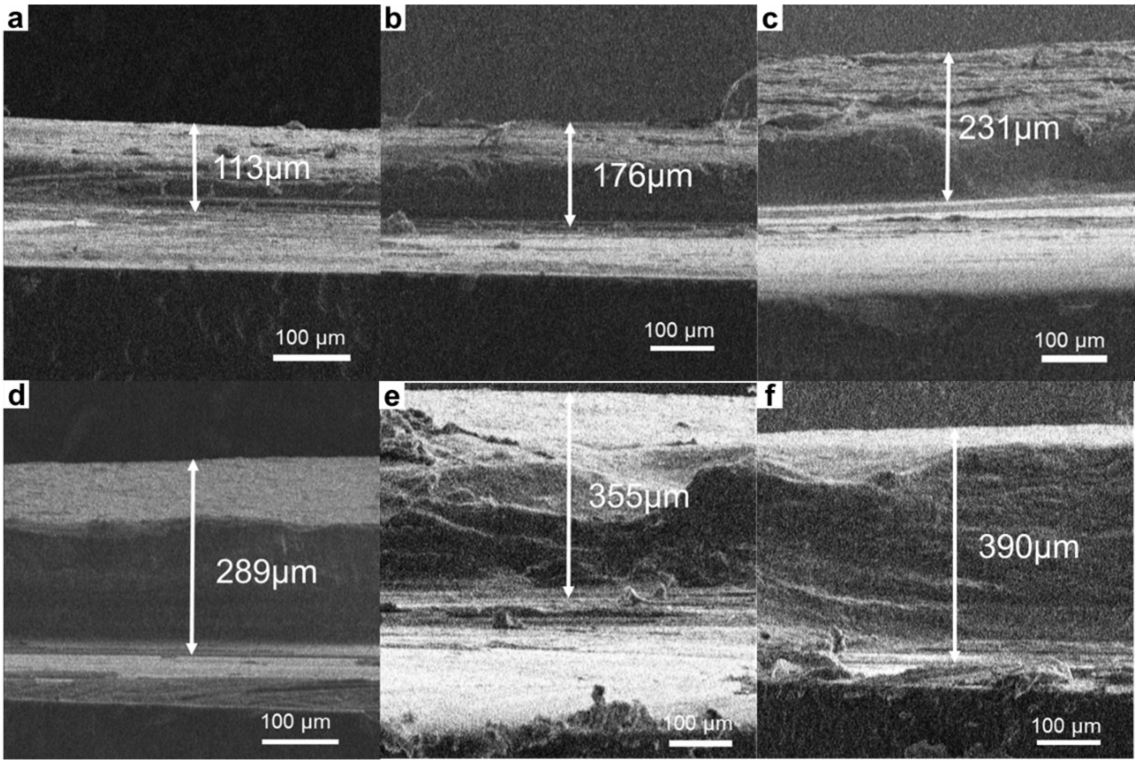

Fig. S9 SEM images for the electrode section of different layer coating (a-f, from one layer to six layers, respectively)

REFERENCES

1. P. T. Kissinger, W. R. Heineman, *J. Chem. Educ.,* 1983, **60**, 702−706.
